# Spiritual Struggle in Parents of Children with Cystic Fibrosis Increases Odds of Depression

**DOI:** 10.1155/2017/5670651

**Published:** 2017-07-31

**Authors:** Rhonda D. Szczesniak, Yuanshu Zou, Sophia M. Stamper, Daniel H. Grossoehme

**Affiliations:** ^1^Division of Biostatistics and Epidemiology, Cincinnati Children's Hospital Medical Center, Cincinnati, OH, USA; ^2^Division of Pulmonary Medicine, Cincinnati Children's Hospital Medical Center, Cincinnati, OH, USA

## Abstract

**Objective:**

Spiritual struggle (SS) is associated with poorer health outcomes including depression. The study's main objectives were to characterize change in depression over time, examine longitudinal associations between SS and depression, and determine the extent to which experiencing SS at baseline was predictive of developing depression at follow-up.

**Methods:**

A two-site study collected questionnaire responses of parents (*N* = 112; 72% female) of children with cystic fibrosis followed longitudinally. Generalized linear mixed effects modeling examined the association between depression and SS over time and assessed potential mediators, moderators, and confounders.

**Results:**

Prevalence of depression increased from baseline to follow-up (OR: 3.6, *P* < 0.0001), regardless of degree of SS. Parents with Moderate/Severe SS were more likely to have depressive symptoms, compared to parents without SS (OR: 15.2, *P* = 0.0003) and parents who had Mild SS (OR: 10.2, *P* = 0.0001). Being female and feeling less “at peace” also significantly predicted increased depression (OR: 2.5, *P* = 0.0397, and OR: 1.15, *P* = 0.0419, resp.). Experiencing SS at baseline was not predictive of having depression subsequently at follow-up.

**Conclusions:**

Parents experiencing SS were significantly more likely to report depressive symptoms. Interventions to reduce SS have shown efficacy and may be considered.

## 1. Introduction

While the overall prevalence of depression in the USA is approximately 10%, it is higher among parents of chronically ill children than in the general population. Depression prevalence rates for parents have been reported at 38% for epilepsy [1] and 33–39% for asthma [2] when their children have these diseases. The prevalence of depression among parents of children with cystic fibrosis (CF) has been reported at 20–35% [3]. Depressive symptoms are associated with poorer adherence by mothers of children with asthma [4] as well as other diseases [5]. Nonadherence to medication adds significant expense to the cost of health care, estimated at over $100 billion per year [6].

Spiritual struggle (SS) refers to “efforts to conserve or transform a spirituality that has been threatened or harmed” [7] and has been reported in 7% to 50% of samples across disease groups [8, 9]. The construct is not inherently negative; it can be resolved in ways that lead to growth or it can become chronic and debilitating [10]. SS has been associated with depression [11, 12], and in a longitudinal study of people who were Orthodox Jews SS preceded and was a potential cause of future depression [13]. SS explained 4% of the variance in depressed mood in a sample of women with breast cancer [11].

CF is a progressive, life-shortening genetic disease with a complex daily treatment regimen. In one study of parents in the first year after their child's CF diagnosis, parents uniformly referred to their experience as “devastating” and described ways in which they reframed their experience in religious or spiritual terms [14]. Given the rates of depression among parents of children with chronic disease and SS among persons with chronic disease, the purpose of this study was to test whether SS was associated with depressive symptoms longitudinally, using parents of children with CF as an exemplar.

## 2. Materials and Methods

### 2.1. Participants and Procedures

Data on SS and depression were collected in the context of a study of parental treatment adherence carried out at the Cystic Fibrosis Centers within two academic pediatric medical centers; one was a 525-bed hospital in the Midwest and the other a 292-bed hospital in the South. The institutional/ethics review boards at both institutions approved this study. Parents who had children who were 13 years of age or younger were notified of the eligibility to participate in a study of parental treatment adherence by a letter from their CF Center Director or directly by study staff in the clinic. Participants were recontacted approximately 2 years later to participate in follow-up data collection, two time points of observation. After completing informed consent, participants were given a link to complete questionnaires using REDCap Survey [15].

#### 2.1.1. Study Measures

*Depression*. The Center for Epidemiologic Studies Depression Scale or CES-D was used to measure depressive symptoms [16]. The outcome of interest, depression, was dichotomized for each participant and time point by using the CES-D score. An indicator variable was created so that scores equal to or exceeding 16 corresponded to clinically significant symptom levels of depression, while scores below 16 corresponded to no depression [16]. Reliability statistics (Cronbach's alpha) for the CES-D and other scales reported in this study have already been reported for the baseline data [17].

*Spiritual Struggle*. SS is commonly operationalized as using negative spiritual coping styles as measured by the Brief R-COPE [18, 19]. The exposure of interest, SS, was defined based on previous studies [8, 19] as follows. The following Fitchett and colleagues [20], responses to the R-COPE items were used to classify SS observed for each participant and time point into one of three levels corresponding to the frequency of negative spiritual coping: “None,” “Mild,” or “Moderate/Severe.” The first category corresponded to a participant not using any form of negative spiritual coping; the second category was for a participant who used all forms of negative spiritual coping only as often as “somewhat”; finally, the last category was for a participant who used any form of negative spiritual coping “quite a bit” or “a great deal.” 

*Spirituality*. The FACIT-Sp was used as a measure of spirituality and well being in the primary study. For the purposes of this study, the “peace” subscale only was retained in order to control for potential relationships between spirituality and depressive symptoms. Higher scores correspond to a greater extent of being “at peace.” The Peace subscale consists of four items (e.g., “I feel a sense of harmony within myself”) for respondents to indicate how true a statement has been for them during the past seven days using a five-point scale ranging from 0 (not at all) to 4 (very much). Reliability for this subscale has been reported at 0.84 [21].

*Demographic and Clinical Information*. Demographic questions created for this study collected data on parent gender, education level, religious affiliation, marital status, child age, and gender. Clinical information related to the child's disease severity was obtained by chart review and consisted of the child's number of pulmonary exacerbations within the year prior to baseline and body mass index percentile.

### 2.2. Statistical Considerations

Descriptive analyses were performed for SS, demographic and clinical characteristics, and the primary outcome, parental depression. Study variables observed at baseline were compared across SS groups (None, Mild, and Moderate/Severe) using Kruskal-Wallis ANOVA for continuous variables and chi-square or Fisher's exact test, as appropriate, for categorical variables. Summaries are reported as *n* (%) for categorical variables and median (IQR) for continuous variables.

Multivariable logistic regression using a generalized linear mixed model with logit link was employed to assess two of the main study objectives. We examined the change in depression from baseline to follow-up by assessing the main effect of time in the model. We examined the nature of the longitudinal association between depression and SS by assessing the interaction between time and SS in the model. This model was examined both without and with adjustment of clinical and demographic covariates, in order to assess the degree of confounding. The mixed model approach was chosen to account for longitudinal correlation and parent dyad effects; random effects were included in the covariance structure of the model for nested repeated measures [22]. Each subject contributed a maximum of two measurements. Differences between the two study sites were examined by including an indicator variable for site in the model fixed effects. Missing data arising from loss to follow-up was accounted for by the likelihood structure in the generalized linear mixed model under the ignorability assumption [23, 24]. SS was treated as a time-varying covariate in the model. Potential confounders were identified from study data collected at baseline and included parental characteristics (gender, religious affiliation, being “at peace,” education level, and having other children with CF) and disease severity characteristics of the child with CF (age, number of exacerbations within the year prior to baseline, and BMI percentile). We assessed the role of confounding on the relationship between SS and depression by including each of the aforementioned variables as covariates in the model. Backward elimination was then performed based on minimizing AIC. The threshold for covariate inclusion during final model selection process was *P* < 0.10.

The third study objective, which was to determine the extent to which experiencing SS at baseline was predictive of depression at follow-up, was examined using a generalized linear mixed model based on cross-sectional data. In this model, depression and SS at baseline were included as fixed effects, while depression status at follow-up was treated as the outcome. Random effects were again incorporated to adjust for parent dyads.

Secondary analyses were performed to assess additional relationships linking SS and depression. First, we assessed the extent to which having depression at baseline was associated with experiencing SS over time using a generalized linear mixed model with multinomial (generalized) logit link. Multinomial regression was performed, given that there were three categories of SS (None, Mild, and Moderate/Severe). SS at baseline was included as a covariate, along with time and the interaction between these two variables. We examined potential confounding similar to the aforementioned approach for covariate selection. Second, we examined the moderating effect of parent gender on the relationship between SS and depression by including an interaction term for parent gender and SS effects in the previously described model. The rationale for this examination was the broad gender differences in spirituality, including the increasing value of spirituality over time for women compared with men, and evidenced that women are more likely than men to experience SS [25, 26]. Third, we examined the potential mediating effects of self-directed spiritual coping, pleading spiritual coping, and emotional support from the congregation on the association between SS and depression using classic mediation analysis with the causal-steps approach [27–30]. These factors were selected because of prior relationships between SS and depressive symptoms [31, 32].

Percentages of concordance and discordance were used to examine the fit of each generalized linear mixed model. Model contrasts were used to assess pairwise differences between SS groups. The OR and 95% confidence interval (CI) are reported; *P* < 0.05 was considered statistically significant for each association of interest corresponding to the main study objectives. All analyses were implemented using SAS 9.3 (SAS Institute, Cary, NC).

## 3. Results

A total of 141 parents (72% female) participated in the baseline data collection (46% participation rate). There were 29 parent dyads that participated in the study. Demographic/clinical information and spiritual characteristics of the cohorts at baseline are provided in Table 1. There were larger percentages of female parents in the Mild and Moderate/Severe SS groups, compared to the percentage of female parents in the group who experienced no SS. The Moderate/Severe SS group had lower median scores for being “at peace,” higher reported levels of depressive symptoms, and a greater percentage meeting criteria for depression, compared to the other study groups. Although the percentage of married participants was high across groups, nearly all of the participants in the group who experienced no SS were married.

Baseline characteristics of parents who discontinued study participation prior to follow-up were compared to characteristics of those parents who remained in the study (Table 2). There were 112 parents (79%) who completed both visits; of those parents who did not have depressive symptoms at the baseline visit, 32 (55%) went on to develop depressive symptoms at follow-up. The association between study completion and religious affiliation approached statistical significance; however, there were no statistically significant differences between subjects based on attrition.

Once model selection was complete, the final longitudinal statistical model for depression included terms for SS, time point (baseline or follow-up), parent gender, and the parent's extent of being “at peace,” which were significant predictors of increased odds of depression. Child age met the threshold of significance for model inclusion. There was no significant interaction between SS and time. Random effects for parent dyads and repeated measures arising from baseline and follow-up were also statistically significant. Site effect was not significant in any of the multivariable models.

Results summarized in Table 3 show that increased SS was associated with higher odds of depression. Parents who exhibited Moderate/Severe SS were more likely to be depressed than their counterparts who had no SS (OR: 15.24; 95% CI: 4.14 to 56.02; *P* = 0.0003). Parents with Moderate/Severe SS were more likely to be depressed than those parents who exhibited Mild SS (OR: 10.17; 95% CI: 2.74 to 37.7; *P* = 0.0001). Those parents with Mild SS had odds of depression that were not significantly different from those who had no SS. Odds of depression were lower at baseline, compared to follow-up. Being a female parent was also associated with increased odds of depression, while being “at peace” corresponded to decreased odds of depression. We also estimated the odds of depression at each level of SS and examined effects over time by fitting a generalized linear mixed model without covariates. Unadjusted analyses also suggest that parents who have Moderate/Severe SS have increased odds of depression, compared to those with Mild SS (OR: 10.81; 95% CI: 3.09 to 37.84; *P* = 0.0005). Findings were similar when comparing those with Moderate/Severe SS and no SS (OR: 19.94; 95% CI: 5.42 to 73.3; *P* < 0.0001). Odds of depression between those with Mild SS and parents who did not exhibit SS were not significantly different.

In secondary analysis with multinomial logistic regression, SS did not change over time when modeled as an outcome variable; however, parents who were depressed at baseline were more likely to experience Mild SS (OR: 20.08, 95% CI: 5.37 to 75.11) or Moderate/Severe SS (OR: 39.95, 95% CI: 9.30 to 171.62), compared to having no SS. This association was consistent over time, as the interaction between baseline depression and time was not statistically significant. Being at peace was retained based on covariate selection in the adjusted model (*P* = 0.0266) and was associated with increased odds of having Mild (OR: 1.07; 95% CI: 0.92 to 1.24) or Moderate/Severe SS (OR: 1.28, 95% CI: 1.07 to 1.55), compared to not experiencing SS. Including the effect of being “at peace” in the adjusted model yielded OR estimates for Mild (OR: 18.62; 95% CI: 4.79 to 72.73) or Moderate/Severe SS (OR: 25.53, 95% CI: 5.78 to 112.73), compared to not experiencing SS, which were slightly lower than in the unadjusted model; however, conclusions for the time effect and its interaction with baseline depression remained statistically nonsignificant.

In other secondary analyses with depression as the outcome, there was no significant interaction between gender and SS. None of the potential mediating effects previously mentioned met criteria for evidence of mediating the effect of SS on depression. Furthermore, having Mild or Moderate/Severe SS at baseline was not predictive of having depression at follow-up. This result was consistent regardless of whether baseline depression status was included as a covariate.

## 4. Discussion

This study shows that a sample of parents of children with CF who experienced SS had increased odds of having depressive symptoms at clinically significant levels. Whether SS precedes or follows depressive symptoms has been an open question. Paragament described three possible direction relationships between SS and depression [13]. First, primary SS could lead to higher psychological distress. Second, primary psychological distress could lead to secondary SS; third, complex SS is described as both the cause and effect of psychological distress. The lack of a significant relationship between SS at baseline in the present study and the development of depression over time tends to support either a secondary or complex struggle model. Pirutinksy and colleagues' study of SS among adults who are Orthodox Jewish was consistent with a primary struggle model. It may be that SS due to worry and stress have a different relationship with distress than do SS due to a life-shortening physical disease. Another possible explanatory factor is that struggles due to one's own condition, rather than a condition of one's child, may lead to a different relationship between SS and distress or depressive symptoms. A clearer understanding of the relationship between SS and distress is needed to guide intervention development. Psychological distress due to primary or complex SS could be ameliorated by an intervention directly addressing SS. Secondary SS could be expected to decrease if the psychological distress is minimized. Conversely, attempts to decrease secondary SS directly may be ineffective.

These results suggest a new avenue to address parental depression and other health outcomes, through addressing parental SS. The inclusion of spirituality into healthcare is of interest to a significant proportion of Americans [33]. The results suggest a collaborative relationship between psychologists and other mental health providers and health care chaplains may be beneficial. Having encountered parents of a child with a chronic illness who is experiencing SS, chaplains may refer the individual to a mental health provider who could screen them for depressive symptoms. Chaplains could also educate psychologists and others to include an SS screening when they work with parents of children with chronic illness. Screening tools could include the negative subscale of the Brief R-COPE as used here, the Religious and Spiritual Struggles Scale [34], or the protocol developed by Fitchett and Risk [9].

SS can be modifiable. Oemig-Dworsky and colleagues demonstrated support for an intervention to reduce SS of college students [35]. Other interventions have been developed and have shown positive results, including one aimed at behavioral change among persons who are HIV+ [36] and sexual-abuse survivors [37]. Tarakeshwar and colleagues developed a spiritual coping intervention for persons with HIV/AIDS [38]. It is therefore reasonable to presume that SS is a modifiable aspect of the lives of parents of children with CF and other chronic diseases that is amenable to intervention with the distal goal of reducing depressive symptoms.

This study had the following limitations. Although depression status changed over time for the cohort, it is not possible to address issues of causality given the observational nature of the study. Having relatively few individuals at baseline that experienced SS while not depressed (Table 1) yielded wide CI for inferential associations between these two attributes. The sample was collected from two CF Centers and comprised of people from seven states in the US; however, all of these were located in the Midwest and South, where religion and spirituality may play a more significant role in people's lives. Therefore, the results may not be representative for CF parents in the USA as a whole. The confidence intervals associated with the odds ratios are somewhat wide; a larger study is needed to increase precision. SS have been described as struggles with the Divine or demonic, as being between persons (interpersonal) as well as within one's self (intrapersonal), whether related to doubt, morality, or meaning [34]. The Brief R-COPE focuses on Divine and, to some extent, intrapersonal struggles. It is not clear how each of these types of SS may contribute to, or be affected by, depressive symptoms. If inter- and intrapersonal SS are more significantly related to depressive symptoms, this could explain why this study did not find that SS was predictive of depression. Future studies may benefit from the tool developed by Exline and colleagues [34], which provides a more nuanced approach to SS. Moderate correlations with depressive symptoms were shown for all subscales showing specific types of SS, with the highest correlation being with the ultimate meaning subscale and depressive symptoms.

## 5. Conclusion

The results of this study could transform the understanding and treatment of depression by opening up a common, salient, and underutilized avenue of intervention and research for chaplains and other psychosocial researchers and clinicians. Given the pervasive prevalence of spirituality and belief in God in North America and relationship of SS to poorer mental health outcomes, this approach is highly likely to be acceptable to many parents of children with CF or other chronic diseases, as well as being an effective means of reducing depression.

## Figures and Tables

**Table 1:** Baseline characteristics of study subjects according to degree of spiritual struggle.

	Degree of spiritual struggle			*P* value^*∗*^
	None (*N* = 55)	Mild (*N* = 56)	Moderate/severe (*N* = 30)	
Parents, *n* (%)				
Female	31 (56.4%)	47 (83.9%)	23 (76.7%)	0.0044
Male	24 (43.6%)	9 (16.1%)	7 (23.3%)	
Religious affiliation, *n* (%)				
Nondenominational Christian	18 (32.7%)	19 (33.9%)	10 (33.3%)	0.47
Protestant	5 (9.1%)	9 (16.1%)	8 (26.7%)	
Roman Catholic	11 (20.0%)	11 (19.6%)	3 (10.0%)	
Other	10 (18.2%)	7 (12.5%)	6 (20.0%)	
None	11 (20.0%)	10 (17.9%)	3 (10.0%)	
Being “at peace,” median (IQR), *n*	10 (8–12), 55	10 (7–12), 55	8.5 (6–11), 30	0.012
Marital status, *n* (%)				
Married	54 (98.2%)	48 (85.7%)	24 (80.0%)	0.009
Not married	1 (1.8%)	8 (14.3%)	6 (20.0%)	
Education level, *n* (%)				
High school (some or completed)	16 (29.1%)	11 (19.6%)	8 (26.7%)	0.38
Some college (did not graduate)	16 (29.1%)	16 (28.6%)	12 (40.0%)	
College/graduate school (completed)	23 (41.8%)	29 (51.8%)	9 (30.0%)	
Multiple children with CF, *n* (%)				
Yes	12 (21.8%)	12 (21.4%)	3 (10.0%)	0.36
No	43 (78.2%)	44 (78.6%)	27 (90.0%)	
CES-D score (depressive symptomology), median (IQR), *n*	9 (4–16), 49	12 (8–24), 49	27 (20–33), 30	<0.0001
Above cutoff for clinically significant depressive symptoms, *n* (%)				
Yes	13 (23.6%)	20 (35.7%)	27 (90.0%)	<0.0001
No	36 (65.5%)	29 (51.8%)	3 (10.0%)	
Child with CF, *n* (%)				
Female	27 (49.1%)	26 (46.4 %)	13 (43.3%)	0.95
Male	27 (49.1%)	29 (51.8%)	15 (50.0%)	
Child age in years, median (IQR), *n*	6 (3–9), 54	7 (3–11), 55	4.5 (3–10), 30	0.52
Pulmonary exacerbations child had in prior year, median (IQR), *n*	0 (0–2), 55	0 (0–2), 56	1 (0–2), 30	0.48
BMI percentile of child, median (IQR), *n*	58 (35–86), 55	56 (31–75), 54	49 (35–86), 30	0.61

*N*, total number of participants at baseline in each category of religious/spiritual struggle; *n*, number of participants with available data; CES-D, Center for Epidemiologic Studies Depression Scale; IQR, intraquartile range; R-COPE, religious coping methods scale; SD, standard deviation; ^*∗*^*P* values were calculated by chi-square test or Fisher's exact test, as appropriate, for categorical variables, and by Kruskal-Wallis test for continuous variables.

**Table 2:** Baseline characteristics of patients by study completion.

	Baseline and follow-up (*N* = 112)	Baseline only (*N* = 29)	*P* Value^*∗*^
Parents, *n* (%)			
Female	80 (71.4%)	21 (72.4%)	0.92
Male	32 (28.6%)	8 (27.6%)	
Religious affiliation, *n* (%)			
Nondenominational Christian	34 (30.4%)	13 (44.8%)	0.14
Protestant	20 (17.9%)	2 (6.9%)	
Roman Catholic	23 (20.5%)	2 (6.9%)	
Other	19 (17.0%)	4 (13.8%)	
None	16 (14.3%)	8 (27.6%)	
Being “at peace,” median (IQR), *n*	10 (8–12), 111	10 (8–12), 29	0.77
Marital status, *n* (%)			
Married	102 (91.1%)	24 (82.8%)	0.20
Not married	10 (8.9%)	5 (17.2%)	
Education level, *n* (%)			
High school (some or completed)	29 (25.9%)	6 (20.7%)	0.15
Some college (did not graduate)	31 (27.7%)	13 (44.8%)	
College/graduate school (completed)	52 (46.4%)	9 (31.0%)	
Multiple children with CF, *n* (%)			
Yes	22 (19.6%)	5 (17.2%)	0.77
No	90 (80.4%)	24 (82.8%)	
CES-D score (depressive symptomology), median (IQR), *n*	12 (8–24), 104	18.5 (6–25), 24	0.71
Above cutoff for clinically significant depressive symptoms, *n* (%)			
Yes	46 (41.1%)	14 (48.3%)	0.21
No	58 (51.8%)	10 (34.5%)	
Child with CF, *n* (%)			
Female	52 (46.4%)	14 (48.3%)	0.99
Male	56 (50.0%)	15 (51.7%)	
Child age in years, median (IQR), *n*	5 (3–11), 111	4 (4–8.5), 28	0.67
Pulmonary exacerbations child had in prior year, median, *n* (IQR)	0 (0–2), 112	1 (0–2), 29	0.15
BMI percentile of child, median (IQR)	59 (34–84), 110	52 (34–66), 29	0.33

^*∗*^ *P* values from Wilcoxon Mann–Whitney test or chi-square test.

**Table 3:** Longitudinal multivariable statistical model examining spiritual struggle and other factors related to depression.

Variable	OR^*∗*^	(95% CI)	*P* value^∧^
Spiritual struggle			
Moderate/severe	15.24	(4.14, 56.02)	0.0003
Mild	1.33	(0.62, 2.82)	
None	1		
Time point			
Baseline	0.15	(0.07, 0.32)	<0.0001
Follow-up	1		
Parent gender			
Female	2.48	(1.05, 5.90)	0.0397
Male	1		
Being “at peace”	0.87	(0.76, 0.99)	0.0419
Child age, in years	0.92	(0.84, 1.01)	0.08

OR, odds ratio; CI, confidence interval. Model also includes adjustment for site, longitudinal correlation, and parent dyad effects (see Materials and Methods). ^*∗*^For categorical variables, the estimate is the odds of having depression being in the indicator group versus the reference group (labeled by OR = 1). For continuous covariates it is the increase in odds of depression when the covariate increased by 1 unit. ^∧^*P* value based on Type III Tests of Fixed Effects.
